# Association between SARS-CoV-2 RNAemia and dysregulated immune response in acutely ill hospitalized COVID-19 patients

**DOI:** 10.1038/s41598-022-23923-1

**Published:** 2022-11-16

**Authors:** Roberta Rovito, Valeria Bono, Matteo Augello, Camilla Tincati, Federica Mainoldi, Guillaume Beaudoin-Bussières, Alexandra Tauzin, Silvia Bianchi, Mohamad Hadla, Vaibhav Yellenki, Antonella d’Arminio Monforte, Stefano Casola, Elisa Borghi, Andrés Finzi, Giulia Marchetti

**Affiliations:** 1 Clinic of Infectious Diseases, Department of Health Sciences, ASST Santi Paolo E Carlo, University of Milan, Via A. di Rudini’ 8, 20142 Milan, Italy; 2 The FIRC Institute of Molecular Oncology (IFOM), via Adamello 16, 20139 Milan, Italy, Italy; 3 Centre de Recherche du CHUM (CRCHUM), Montréal, QC H2X 0A9 Canada; 4 Département de Microbiologie, Infectiologie Et Immunologie, Université de Montréal, Montréal, QC H2X 0A9 Canada; 5 Microbiology and Clinical Microbiology, Department of Health Sciences, ASST Santi Paolo E Carlo, University of Milan, Via A. di Rudinì 8, 20142 Milan, Italy, Milan, Italy

**Keywords:** Viral infection, Viral infection

## Abstract

Severe/critical COVID-19 is associated with immune dysregulation and plasmatic SARS-CoV-2 detection (i.e. RNAemia). We detailed the association of SARS-CoV-2 RNAemia with immune responses in COVID-19 patients at the end of the first week of disease. We enrolled patients hospitalized in acute phase of ascertained SARS-CoV-2 pneumonia, and evaluated SARS-CoV-2 RNAemia, plasmatic cytokines, activated/pro-cytolytic T-cells phenotypes, SARS-CoV-2-specific cytokine-producing T-cells (IL-2, IFN-γ, TNF-α, IL-4, IL-17A), simultaneous Th1-cytokines production (polyfunctionality) and amount (iMFI). The humoral responses were assessed with anti-S1/S2 IgG, anti-RBD total-Ig, IgM, IgA, IgG1 and IgG3, neutralization and antibody-dependent cellular cytotoxicity (ADCC). Out of 54 patients, 27 had detectable viremia (viremic). Albeit comparable age and co-morbidities, viremic more frequently required ventilatory support, with a trend to higher death. Viremic displayed higher pro-inflammatory cytokines (IFN-α, IL-6), lower activated T-cells (HLA-DR+CD38+), lower functional SARS-CoV-2-specific T-cells (IFN-γ+CD4+, TNF-α+CD8+, IL-4+CD8+, IL-2+TNF-α+CD4+, and IL-2+TNF-α+CD4+ iMFI) and SARS-CoV-2-specific Abs (anti-S IgG, anti-RBD total-Ig, IgM, IgG1, IgG3; ID_50_, %ADCC). These data suggest a link between SARS-CoV-2 RNAemia at the end of the first stage of disease and immune dysregulation. Whether high *ab initium* viral burden and/or intrinsic host factors contribute to immune dysregulation in severe COVID-19 remains to be elucidated, to further inform strategies of targeted therapeutic interventions.

## Introduction

COVID-19 clinical phenotype is highly heterogeneous, from flu-like symptoms to life-threatening multiorgan dysfunction and death^1^, with severe and critical COVID-19 in 14% and 5% of infected individuals, respectively^2^. Clinical aggravation occurs within a median of 8 (7–14) days after disease onset^2^, which corresponds to the temporal bridging between innate and adaptive immunity.

Immunoprofiling of acute severe COVID-19 described hyperactivated/exhausted T-cells, together with fewer circulating follicular helper T-cells (cTfh) and plasmablasts (PBs)^3–6^. On the other side, the presence of SARS-CoV-2 specific T-cells was associated with a milder COVID-19 disease^7^, whereas the levels of neutralizing antibodies do not necessarily correlate with severity^8,9^. As a delayed IFNs responses have been described in SARS-CoV-2 infection^10^, it is plausible that the innate immune response compensates the delay of the adaptive immunity by means of hyperactivation, in turn leading to a pro-inflammatory environment and immunopathology^11^.

Despite several factors have been associated to worse disease outcome, that include older age, cardiovascular co-morbidities, diabetes, and immune depression^1,2^, the mechanisms underlying the immune dysregulation dictating worse disease outcome still lack a detailed definition, and are most likely characterized by a complex virus-host interplay. Among viral factors, similar to other respiratory viruses^12,13^, SARS-CoV-2 RNA has been detected in the blood of some patients^14,15^ as early as the first week from disease onset^14,16^, and has been associated with a pro-inflammatory host response, tissue damage^17,18^, disease progression and death^19^. Whether such circulating SARS-CoV-2 RNAs represent infectious particles still lacks full clarification, yet the presence of circulating virions has been recently demonstrated in COVID-19 patients^20^. The source of SARS-CoV-2 RNAemia is not entirely understood, most likely occurs via leakage from damaged lung tissues and direct endothelial cells infection. SARS-CoV-2 genome can be found in tissues such as liver, spleen, heart and intestine^21–23^, confirming that COVID-19 pathogenesis involves extrapulmonary tissues. Additionally, viremia has been described as a major player in transplacental SARS-CoV-2 transmission^24^. Therefore, the dissemination of SARS-CoV-2 into the bloodstream may represent a critical step in COVID-19 pathogenesis to further drive multiorgan failure.

While specific immunoprofiles and SARS-CoV-2 RNAemia, have both been individually associated with disease severity, to date, a major knowledge gap exists on the thorough understanding of how much of the COVID-19 immune dysregulation is ascribable to viral factors. We hereby aimed to investigate the associations between SARS-CoV-2 RNAemia, referred to as viremia, and immune changes in hospitalized COVID-19 patients during the acute phase of disease.

## Results

### Participants characteristics and SARS-CoV-2 viremia

54 hospitalized COVID-19 patients were included: 27/54 with detectable SARS-CoV-2 viremia (2.2, 1.3–5.4 log_10_RNA copies/ml). Demographic and clinical characteristics of the study population are summarized in Table 1. Interestingly, despite comparable for age, sex, co-morbidities, frequency and duration of symptoms (7, 4–10 days), and COVID-19 treatments, viremic patients displayed a more severe disease: a higher proportion of patients needing Continuous Positive Airway Pressure (CPAP), Non-Invasive Ventilation (NIV) or Oro-Tracheal Intubation (OTI) (23, 85.2% vs. 15, 55.6%; p = 0.035], and a trend to higher proportion of deaths (9, 33.3%, vs. 3, 11.1%; p = 0.099, respectively). Additionally, SARS-CoV-2 viremia inversely correlated with the PaO_2_/FiO_2_ *nadir*, well-known marker of disease severity (r = − 0.3, p = 0.02) (Supplementary Fig. S1).

**Table 1:** Demographic and clinical characteristic of study subjects.

Demographic and clinical characteristics	All COVID-19 patients (n = 54)	Viremic COVID-19 patients (n = 27)	Aviremic COVID-19 patients (n = 27)	*p*-value viremic vs. aviremic COVID-19 patients
**Sex, n (%)**				
Male	39 (72.2)	20 (74.1)	19 (70.4)	> 0.9999
Female	15 (27.8)	7 (25.9)	8 (29.6)	
**Age, years, Median (IQR)**	64 (52.50–75.25)	67 (55–78)	63 (45–71)	0.1659
**Ethnicity, n (%)**				
Caucasian	46 (82.2)	24 (88.9)	22 (81.5)	0.7040
Maghrebi/Middle Eastern	1 (1.9)	1 (3.7)	0 (0)	> 0.9999
South Asian	1 (1.9)	1 (3.7)	0 (0)	> 0.9999
Latin American	6 (11.1)	1 (3.7)	5 (18.5)	0.1917
**Comorbidities, n (%)**				
Any comorbidity	29 (53.7)	16 (59.3)	13 (48.2)	0.5857
Hypertension	20 (37)	10 (37)	10 (37)	> 0.9999
Myocardial infarction	8 (14.8)	6 (22.2)	2 (7.4)	0.2501
Cardiac arrhythmia	3 (5.6)	1 (3.7)	2 (7.4)	> 0.9999
COPD	1 (1.9)	1 (3.7)	0 (0)	> 0.9999
Asthma	2 (3.7)	0 (0)	2 (7.4)	0.4906
Peptic ulcer	2 (3.7)	0 (0)	2 (7.4)	0.4906
Chronic kidney disease	2 (3.7)	2 (7.4)	0 (0)	0.4960
Diabetes	14 (25.9)	9 (33.3)	5 (18.5)	0.3520
Stroke	3 (5.6)	1 (3.7)	2 (7.4)	> 0.9999
Dementia	1 (1.9)	0 (0)	1 (3.7)	> 0.9999
Cancer	2 (3.7)	2 (7.4)	0 (0)	0.4960
Rheumatologic disease	1 (1.9)	0 (0)	1 (3.7)	> 0.9999
**BMI, Median (IQR)**	26.70 (23.98–29.45)	26.40 (25.70–27.14)	27.10 (22.95–31.73)	0.7759
**Smoke, n (%)**				
Current smoker	2 (3.7)	1 (3.7)	1 (3.7)	> 0.9999
Former smoker	12 (22.2)	7 (25.9)	5 (18.5)	0.7445
Never smoker	14 (25.9)	3 (11.1)	11 (40.7)	0.0276*
Unknown	26 (48.1)	16 (59.3)	10 (37)	
**Symptoms at admission, n (%)**				
Fever	51 (94.4)	26 (96.3)	25 (92.6)	> 0.9999
Arthromyalgia	7 (13)	3 (11.1)	4 (14.8)	> 0.9999
Chest pain	7 (13)	1 (3.7)	6 (22.2)	0.1003
Cough	33 (61.1)	16 (59.3)	17 (63)	> 0.9999
Dyspnea	37 (68.5)	18 (66.7)	19 (70.4)	> 0.9999
Gastrointestinal symptoms	16 (29.6)	8 (29.2)	8 (29.6)	> 0.9999
Nausea/vomiting	5 (9.3)	3 (11.1)	2 (7.4)	> 0.9999
Abdominal pain	1 (1.9)	0 (0)	1 (3.7)	> 0.9999
Diarrhea	13 (24.1)	7 (25.9)	6 (22.2)	> 0.9999
Syncope	3 (5.6)	1 (3.7)	2 (7.4)	> 0.9999
Headache	1 (1.9)	0 (0)	1 (3.7)	> 0.9999
Anosmia/dysgeusia	10 (18.5)	6 (22.2)	4 (14.8)	0.7277
**Duration of symptoms before hospitalization, days, Median (IQR)**	7 (4–10)	7 (4–10)	6 (4–14)	0.4099
**Radiological pulmonary infiltrates, n (%)**				
Bilateral	50 (92.6)	25 (92.6)	25 (92.6)	> 0.9999
Unilateral	4 (7.4)	2 (7.4)	2 (7.4)	
**Blood exams upon admission, Median (IQR)**				
Hemoglobin, g/dL	13.60 (12.18–15)	14 (11.80–15.30)	13.30 (12.20–14.60)	0.2005
Leucocytes, 10^3^/μL	6.62 (5.47–8.64)	7.01 (5.73–8.87)	6.02 (5.04–8.56)	0.4679
Neutrophils, 10^3^/μL	4.94 (4.13–7.08)	5.10 (4.38–7.51)	4.50 (3.07–7.04)	0.2763
Lymphocytes, 10^3^/μL	1.02 (0.64–1.38)	1 (0.59–1.35)	1.04 (0.67–1.38)	0.2998
Monocytes, 10^3^/μL	0.44 (0.33–0.59)	0.41 (0.31–0.54)	0.48 (0.33–0.73)	0.2877
Platelets, 10^3^/μL	209 (159.80–252.50)	198 (150–231)	215 (164–268)	0.2468
Creatinine, mg/dL	0.95 (0.70–1.20)	1.10 (0.80–1.20)	0.80 (0.60–1.10)	0.0283*
AST, U/L	43 (34.75–61.25)	43 (35–53)	43 (32–65)	0.9078
ALT, U/L	32 (23–56.75)	32 (25.25–50.50)	32 (23–76.50)	0.6662
CRP, mg/L	69.55 (45.58–112.20)	80.30 (53.20–121.90)	68.70 (40–102)	0.1624
Ferritin, ng/mL	629 (239.50–1115)	642 (269.50–1235)	535 (225–1115)	0.6623
D-dimer, ng/mL	378.50 (258.50–764.30)	460 (236–822.80)	361.50 (272.50–878)	0.8112
LDH, U/L	301 (244.30–393.80)	341 (277.50–389.80)	286.50 (221.30–400.80)	0.1926
CPK, U/L	85 (47.50–173.30)	118.50 (57.50–251.80)	67.50 (36–119.50)	0.0133*
**Maximum oxygen therapy, n (%)**				
None	6 (11.1)	1 (3.7)	5 (18.5)	0.1917
Low/high-flow systems	10 (18.5)	3 (11.1)	7 (25.9)	0.2935
CPAP/NIV/OTI	38 (70.4)	23 (85.2)	15 (55.6)	0.0352*
**Medical therapy, n (%)**				
LPV/r or DRV/c	16 (29.6)	9 (33.3)	7 (25.9)	0.7664
Hydroxychloroquine	41 (75.9)	19 (70.4)	22 (81.5)	0.5256
Azithromycin	18 (33.3)	8 (29.6)	10 (37)	0.7734
Remdesivir	2 (3.7)	2 (7.4)	0 (0)	0.4960
Biologic drugs	12 (22.2)	7 (25.9)	5 (18.5)	0.7445
Steroids	20 (37)	12 (44.4)	8 (29.6)	0.3983
Celecoxib	7 (13)	6 (22.2)	1 (3.7)	0.1003
Interferon beta 1a	1 (1.9)	1 (3.7)	0 (0)	> 0.9999
Heparin prophylaxis	41 (75.9)	21 (77.8)	20 (74.1)	> 0.9999
Hyperimmune plasma	3 (5.6)	3 (11.1)	0 (0)	0.2358
**Duration of hospitalization, days, Median (IQR)**	14.50 (6–25)	20 (8–28)	13 (6–22)	0.4258
**Outcome, n (%)**				
Death	12 (22.2)	9 (33.3)	3 (11.1)	0.0994
Dismissal	42 (77.8)	18 (66.7)	24 (88.9)	

*COPD:* chronic obstructive pulmonary disease; *BMI:* body mass index; *P*_*a*_*O*_*2*_ arterial partial pressure of oxygen; *F*_*i*_*O*_*2*_ fraction of inspired oxygen; *AST:* aspartate aminotransferase; *ALT:* alanine aminotransferase; *CRP:* C reactive protein; *LDH:* lactate dehydrogenase; *CPK:* creatine phosphokinase; *CPAP:* continuous positive airway pressure; *NIV:* noninvasive ventilation; *OTI:* orotracheal intubation; *LPV/r:* lopinavir/ritonavir; *DRV/c:* darunavir/cobicistat. Fisher exact test or Mann–Whitney *U* test; *IQR:* interquartile range. *Statistical significance at *p*-value.

### Plasmatic cytokines

**Figure 1:**
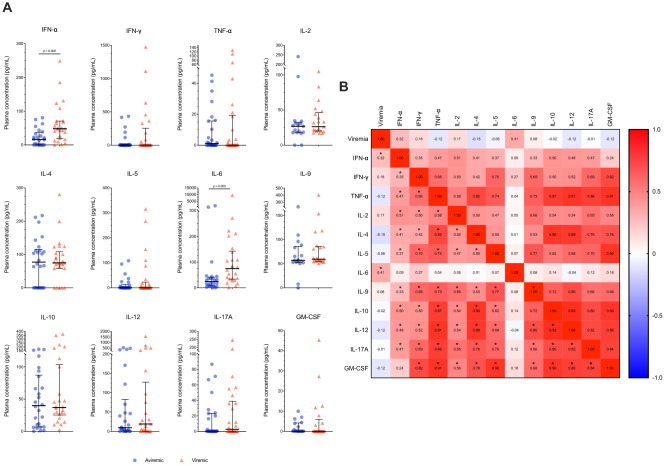
**Plasmatic cytokines in COVID-19 patients according to SARS-CoV-2 viremia.** (**A**) Plasmatic cytokines in aviremic and viremic COVID-19 patients. IFN-α (n = 24 and n = 26), IFN-γ (n = 26 and n = 25), IL-2 (n = 17 and n = 21), IL-4 (n = 26 and n = 26), IL-5 (n = 26 and n = 26), IL-6 (n = 24 and n = 26), IL-9 (n = 17 and n = 21), IL-10 (n = 26 and n = 26), IL-12p70 (n = 26 and n = 25), IL-17A (n = 25 and n = 26), TNF- α (n = 26 and n = 26), GM-CSF (n = 17 and n = 21). (**B**) Heatmap of correlations between SARS-CoV-2 viremia and plasmatic cytokines. Median and interquartile range (IQR) are shown for each group of patients. Mann–Whitney *U* test and Spearman’s correlation test, *statistical significance at p-value < 0.05.

Plasma cytokine profile revealed that compared to aviremic, viremic patients showed a significantly higher plasmatic concentration of IL-6 (74.31, 33.8–142.3, vs. 24.36 pg/ml, 7.7–41.8; p = 0.003) and IFN-α (48.1, 19.2–70.85, vs. 16.27 pg/ml, 0–37.6; p = 0.002), positively correlating with SARS-CoV-2 viremia (Spearman r = 0.32 and 0.41; p = 0.022; 0.003, respectively) (Fig. 1A,B).

### T-cell immunophenotype

**Figure 2:**
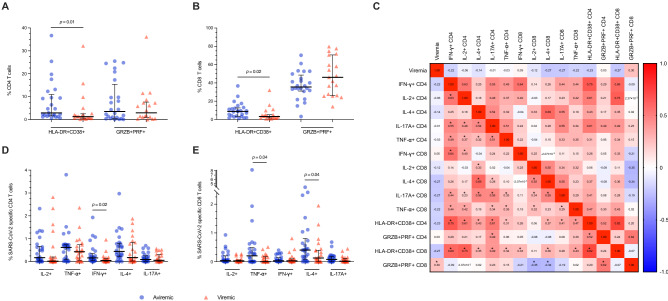
**Immunophenotypes and SARS-CoV-2-specific T cells in COVID-19 patients according to SARS-CoV-2 viremia.** (**A**) Frequencies of circulating HLA-DR + CD38 + (activated) and GRZB + PRF + (pro-cytolitic) CD4 T cells in aviremic and viremic COVID-19 patients (n = 25 and n = 21). (**B**) Frequencies of circulating HLA-DR + CD38 + (activated) and GRZB + PRF + (pro-cytolitic) CD8 T cells in aviremic and viremic COVID-19 patients (n = 25 and n = 21). (**C**) Heatmap of correlations between SARS-CoV-2 viremia, immunophenotypes, and SARS-CoV-2-specific T cells. (**D**) Frequencies of CD4 T cells producing cytokines upon PBMCs challenge for 5 h with 1 μg/ml of a pool of 15-mer peptides of SARS-CoV-2 S, N- and M-proteins in aviremic and viremic COVID-19 patients (n = 19 and n = 17). (**E**) Frequencies of CD8 T cells producing cytokines upon PBMCs challenge for 5 h with 1 μg/ml of a pool of 15-mer peptides of SARS-CoV-2 S-, N- and M-proteins in aviremic and viremic COVID-19 patients (n = 19 and n = 17). Median and interquartile range (IQR) are shown for each group of patients. Mann–Whitney *U* test and Spearman’s correlation test, *statistical significance at p-value < 0.05.

Having shown heightened pro-inflammatory milieu in viremic COVID-19 patients, we next sought to asses T-lymphocyte activation and pro-cytolytic phenotypes. Despite similar proportion of total CD4 + and CD8 + T-cells, viremic patients showed a significantly lower frequency of activated HLA-DR + CD38 + CD4 + (1.26%, 0.6–2.5, vs. 2.9%, 1.58–10.94; p = 0.01) (Fig. 2A) and CD8 + (3.26%, 1.82–5.21, vs. 8.86, IQR: 3.48–12.73; p = 0.02) (Fig. 2B). Interestingly, the viremic group showed a non-significant trend towards higher pro-cytolytic GRZB + PRF + CD8 + (46.1%, 26.31–70.6, vs. 35.5%, 31.05–48.52; p = 0.129) (Fig. 2B), positively correlating with viremia (Spearman r = 0.3, p = 0.05) (Fig. 2C).

### SARS-CoV-2-specific T-cell response

To assess whether reduced activated T-cells in viremic patients also reflected a reduced SARS-CoV-2-specific T-cell response, intracellular cytokines production upon stimulation with 3 pooled SARS-CoV-2 peptides (i.e. S-, N- and M) was analyzed. A trend towards reduced % of SARS-CoV-2-specific cytokine-producing CD4 + was observed in viremic patients, reaching statistical significance for IFN-γ + CD4 + (0.05%, 0.01–0.13, vs. 0.16%, 0.05–0.36; p = 0.022) (Fig. 2D), with a negative, yet non-significant, correlation with SARS-CoV-2 viremia (Spearman r = − 0.22, p = 0.131) (Fig. 2C). Similarly, reduced SARS-CoV-2-specific cytokine-producing CD8 + were observed in the viremic group, reaching significance for TNF-α + (0%, 0–0.17, vs. 0.2%, 0–0.5; p = 0.042) and IL-4 + CD8 + (0.13%, 0–0.4, vs. 0.4%, 0.1–0.8; p = 0.04) (Fig. 2E) with a trend towards a negative correlation with viremia (Spearman r = − 0.22; − 0.27, p = 0.133; 0.061, respectively) (Fig. 2C).

**Figure 3:**
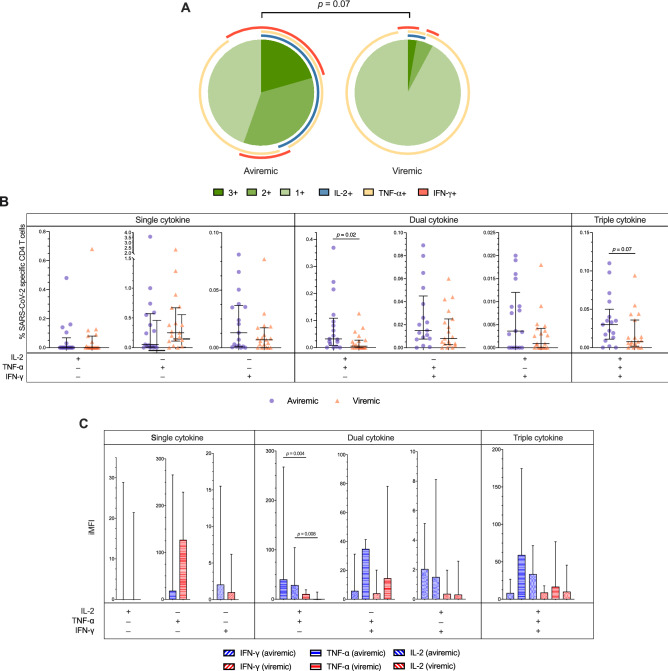
**SARS-CoV-2 specific CD4 Th1 cells functionality in COVID-19 patients according to SARS-CoV-2 viremia.** (**A**) Pie charts showing the median distribution of polyfunctionality profiles in SARS-CoV-2–specific cytokine-producing Th1 cells of aviremic and viremic individuals. The pie slices represent median percentages of tri- (3 +), bi- (2 +), and mono- (1 +) functional T-cells. The arches around the circumference indicate the cytokine (IFN-g, TNF-a, or IL-2) produced by the portion of T-cells that lie under the arc; parts of the pie surrounded by multiple arches represent polyfunctional cells. Statistics: permutation test performed by SPICE 6.0. (**B**) Th1 polyfunctionality: frequencies of CD4 T cells producing one Th1 cytokine (Single cytokine), two Th1 cytokines (Dual cytokine), or three Th1 cytokines (Triple cytokine) upon PBMCs challenge for 5 h with 1 μg/ml of a pool of 15-mer peptides of SARS-CoV-2 S-, N- and M-proteins in aviremic and viremic COVID-19 patients (n = 19 and n = 17). (**C**) Magnitude of Th1 cytokines produced: the integrated median intensity fluorescence (iMFI) was calculated by multiplying the frequency of Single cytokine, Dual cytokine or Triple cytokine SARS-CoV-2-specific Th1 cells with the MFI for a given cytokine in aviremic and viremic COVID-19 patients (n = 19 and n = 17). Statistics: Mann–Whitney *U* test performed by SPICE 6.0.

To further assess whether the reduced % of SARS-CoV-2-specific CD4 + in viremic patients reflected reduced function, the Th1 polyfunctionality was assessed by means of SPICE analysis. A trend towards a different distribution of polyfunctional profiles was observed between groups, as well as reduced percentages of tri- and bi-functional Th1 CD4 + reaching significance for IL-2 + TNF-α + CD4 + (0.005%, 0–0.03 vs. 0.03%, 0.01–0.11; p = 0.03) (Fig. 3A,B). Both tri- and bi-functional CD4 + Th1 cells showed a trend towards negative correlations with viremia, which reached statistical significance for IL-2 + TNF-α + CD4 + (Spearman r = − 0.4, p = 0.017) (Supplementary Fig. 2A).

To further profile polyfunctional Th1 CD4 + , the quantity of cytokine production was investigated by iMFI, showing a trend towards a reduced iMFI of the cytokines produced by bi- and tri-functional Th1CD4 + in viremic patients, reaching statistical significance for IL-2 and TNF-α produced by IL-2 + TNF-α + CD4 + (p = 0.004; 0.008, respectively) (Fig. 3C), and negatively correlating with viremia (Spearman r = − 0.49 and − 0.43, p = 0.002; 0.009, respectively) (Supplementary Fig. 2B).

Next, in order to assess whether the reduced CD4 + Th1 functionality in the viremic group was instead due to a Th2 or Th17 skewing, IL-4 and IL-17a production was further characterized. No differences in the % of IL-4 or IL-17A-producing tri- and bi-functional Th1 cells was found between groups (Supplementary Fig. 3A,B).

In the search of differences in CD4 + and CD8 + response towards individual S-, N- or M-peptides, we found no differences in the % of S- and N-specific CD4 + between groups (Supplementary Fig. 4A,B). However, the % of M-specific IL-4 + and IL-17A + CD4 + was higher in viremic (p = 0.023; 0.029, respectively) (Supplementary Fig. 4C). No significant differences in the % of S-, N- or M- specific CD8 + were found between groups (Supplementary Fig. 4A,C).

### SARS-CoV-2-specific humoral response

**Figure 4:**
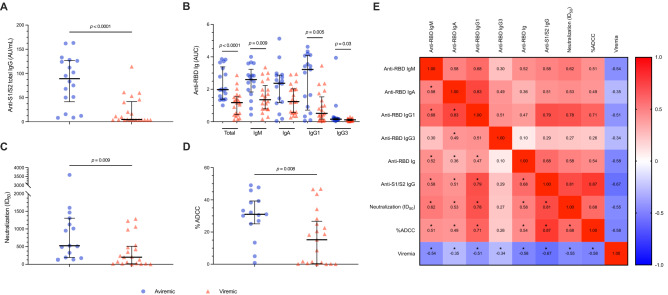
**Elicitation and functionality of S1/S2- and RBD-specific antibodies in COVID-19 patients according to SARS-CoV-2 viremia.** (**A**) Anti-S1/S2 IgG (AU/ml) were assessed by means of a commercially available ELISA (LIAISON SARS-CoV-2 S1/S2 IgG, DiaSorin) in aviremic and viremic COVID-19 patients (n = 18 and n = 22). (**B**) Anti-RBD Ig (AUC) were assessed in aviremic and viremic COVID-19 patients by means of an in-house ELISA (n = 18 and n = 22 for total Ig; n = 17 and n = 21 for IgM, IgA, IgG1 and IgG3). (**C**) Neutralization activity was measured by incubating pseudoviruses bearing the D614G SARS-CoV-2 Spike with serial dilutions of plasma for 1 h at 37 °C before infecting 293 T-ACE2 target cells. Neutralization half maximal inhibitory serum dilution (ID_50_) values were determined using a normalized non-linear regression. (**D**) %ADCC in the presence of plasma at a 1/500 dilution. CEM.NKr parental cells were mixed at a 1:1 ratio with CEM.NKr-Spike cells and were used as target cells. PBMCs from uninfected donors were used as effector cells in a FACS-based ADCC assay. (**E**) Heatmap of correlations between SARS-CoV-2 viremia and humoral response. Median and interquartile range (IQR) are shown for each group of patients. S1/S2-specific total IgG, RBD-specific total Abs, IgM, IgA, IgG1 and IgG3 were negatively correlated with SARS-CoV-2 viremia (Spearman r = − 0.67, − 0.58, − 0.54, − 0.35, − 0.51 and − 0.34; p-value = 0.000003, 0.0001, 0.0005, 0.032, 0.001, 0.04, respectively). Mann–Whitney *U* test and Spearman’s correlation test, *statistical significance at p-value < 0.05.

Having shown an association between viremia and impaired SARS-CoV-2-specific CD4 + functionality we next assessed whether this was proportional to a lower humoral response. Viremic patients displayed significantly lower S1/S2-specific IgG (4.53, 3.8–41.7 vs. 89.15 AU/ml, 41.45–126.5]; p < 0.0001) and total RBD-specific Abs (1.18, 0.44–1.54 vs. 1.98 AUC, 1.39–3.38; p < 0.0001), with a similar trend in RBD-specific isotypes and subclasses, with IgG3 barely produced in both groups (Fig. 4A,B), both of which negatively correlated with viremia (Fig. 4E).

Finally, in order to evaluate whether the lower humoral response in the viremic group was proportional to reduced functionality, the capacity of plasma to neutralize pseudoviral particles carrying the SARS-CoV-2 S D614G glycoprotein or to induce Fc-mediated effector functions were measured. A lower neutralizing activity (200.5, 16.93–509.2, vs. 526.9, 193.6–1305; p = 0.009) (Fig. 4C), as well as a lower % of ADCC activity (15.24, 0.38–26.6)] vs. 31.02, 25.08–39.25; p = 0.008), were observed in viremic patients (Fig. 4D). Both the neutralization and %ADCC negatively correlated with SARS-CoV-2 viremia (Spearman r = − 0.55; − 0.58, p = 0.0005; 0.0002, respectively) (Fig. 4E), suggesting a constrained SARS-CoV-2 specific humoral immunity in viremic COVID-19 patients.

## Discussion

In acutely ill hospitalized COVID-19 patients, we hereby assessed the immune response *vis-à-vis* the detection of SARS-CoV-2 viremia showing that, albeit similar for age, co-morbidities and symptoms’ duration, patients with detectable viremia present a dysregulated immunity at the end of the first week of disease, that is not seen in aviremic individuals. In particular, viremic patients show a pro-inflammatory cytokine signature, lower activated T-cells, less functional SARS-CoV-2-specific T-cells and Abs.

A positive correlation of SARS-CoV-2 viremia with plasma IL-6 was observed, in line with previous findings^15^, as well as with IFN-α. Despite SARS-CoV-2 is a poor type I IFNs inducer^10,25^, in vitro studies showed that, despite similar pro-inflammatory cytokine and chemokine signatures, high-multiplicity of infection (MOI) leads to high levels of IFNs compared to low-MOI^10^. Additionally, an upregulation of interferon-stimulated genes and various inflammatory pathways in immune cells containing SARS-CoV-2 RNA was recently shown^26^. As both IFN-α and IL-6 are PAMP-triggered inflammatory cytokines, the presence of SARS-CoV-2 RNA in the bloodstream may reflect a heightened immune exposure to PAMPs, adding to the already amplified pro-inflammatory signature featuring acutely ill COVID-19 patients.

Peripheral blood immunoprofiling revealed a constricted pool of activated HLA-DR + CD38 + T-cells in viremic patients that occurs in the backdrop of similar clinical and laboratory findings, suggesting that such changes are not due to pre-existing conditions nor to different cell numbers. Whereas several studies have shown a hyperactivated T-phenotype in COVID-19 patients subjects compared to healthy controls, as well as in severe COVID-19 individuals compared to mild, fewer data exist on the association between such phenotype and SARS-CoV-2 viremia^3,5^. Therefore, the underlying mechanisms by which viremic patients showed less circulating activated T cells remain to be elucidated. Moreover, the magnitude of SARS-CoV-2-specific T-cells producing any of the cytokines that we assessed, i.e. IFN-γ, TNF-α, IL-2, IL-4 or IL-17A, was lower in viremic patients, and was more evident in the CD4 + subset. Interestingly, when considering the response to individual peptides, i.e. S or N or M, viremic individuals developed a dominant Th2 and Th17 CD4 response against the M protein. Whereas the Th2/Th1 and Th17 imbalance is a sign of a dysfunctional immune response because associated with disease severity^27,28^, the role of developing a dominant M-specific response over a S- or N-specific response is still controversial. Indeed, while some authors did not find an association^29^, others have described a higher M/NP-specific multifunctional CD8 response compared to spike-specific T cells in mild cases but not in severe COVID-19 patients^30^. Overall, considering that the majority of COVID-19 individuals develop S-, N- and M-specific responses^31^, altogether these data may suggest a role of the type of response over the type of viral antigen in the pathogenesis of COVID-19, that need further clarification. Importantly, similarly to other infections, the reduced T-cell magnitude does not necessarily reflect reduced functionality. In HIV infection, for instance, whereas the magnitude of T-cell response did not necessarily correlate with disease progression^32,33^, higher proportion of polyfunctional CD4 + featured long-term non-progressors, suggesting a better control of the infection^34,35^. In a murine model of *L. Major*, polyfunctional T-cells strongly correlated with protection against re-challenge, to appoint T-cell response quality as major correlate of protection^36^. Furthermore, because polyfunctional T-cells also feature increased cytokine production by MFI in several viral settings^37^, the reduced pool of polyfunctional CD4 + with lower iMFI in our viremic COVID-19 cohort, altogether suggest scant functional potential within the CD4 + pool, which on a per-cell basis, also produce less cytokines.

COVID-19 severity has been associated to an extrafollicular B-cell activation, reduced cTfh, robust Tbet^+^ PB response and early production of high levels of SARS-CoV-2 specific neutralising Abs^6,38,39^, suggesting a T-cell independent B-cell response. In our cohort, we expand the knowledge on the negative correlation between SARS-CoV-2-specific Abs and SARS-CoV-2 viremia^15^ by also describing its association with both the neutralization capacity of killing the virus outside the cell, and Fc-effector functions induction. Our findings well fit with both the described association between an impaired Abs capability to induce Fc-mediated effectors functions and COVID-19 mortality^15,40^, and animal data whereas linking humoral function impairment with lack of control over viral load reduction and SARS-CoV-2 infection^41,42^.

While our study confirms the critical role of SARS-CoV-2 viremia in COVID-19 pathogenesis by showing an aggravated clinical course in viremic patients, the exact meaning of SARS-CoV-2 viremia, i.e. how it occurs, whether it reflects the presence of viable virions and how impacts immunity are still largely matter for scientific debate, and cannot be established in the present study. However, by designing the associations between SARS-CoV-2 viremia and multi-layered adaptive immunity in acute COVID-19, our study identifies at least two non-mutually exclusive pathogenetic models. Firstly, an excessively heightened SARS-CoV-2 viremia ab initio might impair the potential for a functional adaptive immunity, as it has been shown in murine models of lymphocytic choriomeningitis virus where higher viral loads and greater epitopes presentation led to severe T-cells exhaustion^43^. Secondly, a defective immune response in the first disease phase might as well lead to the establishment of SARS-CoV-2 viremia. For instance, a poor early innate response against SARS-CoV-2, e.g. due to genetic factors, which is associated to severe/fatal COVID-19 may lead to an uncontrolled viral replication^11,44^ that eventually may induce viremia.

Lastly, along with the growing pipeline of effective antiviral and monoclonal antibody therapies, our data urge a thorough scrutiny of their efficacy according to detectable SARS-CoV-2 viremia to inform the best put in place of targeted therapeutic strategies^45^.

## Methods

### Study population

We consecutively enrolled patients with ascertained acute COVID-19 (positive RT-PCR nasopharyngeal swab), and radiologically documented pneumonia hospitalized at the Clinic of Infectious Diseases and Tropical Medicine, University of Milan, ASST Santi Paolo e Carlo, Italy, between March and September 2020. This study was approved by the Institutional Ethics Committee (Comitato Etico ASST Santi Paolo e Carlo; 2020/ST/049, 2020/ST/049_BIS, 11/03/2020); written informed consent was obtained from participants. All research was performed in accordance with the Declaration of Helsinki.

### Plasmatic SARS-CoV-2 RT-qPCR

Viral RNA was extracted from 140 ml of thawed plasma by using the QIAamp Viral RNA Mini Kit (QIAGEN), and quantified by real-time PCR using the CDC 2019-nCoV_N1 primers and probe set (Centers for Disease Control and Prevention, Update June 2020) and the TaqPath^™^ 1-Step RT-qPCR Master Mix CG (ThermoFisher). The 2019-nCoV_N Positive Control plasmid (Integrated DNA Technologies, Inc.) was used for absolute quantification, a non-template condition was used as negative control, and the *RPP30* quantification for RNA extraction quality assessment. The assay was run in duplicate.

### Multiple detection of cytokines

Plasmatic cytokines (IFN-α, IFN-γ, IL-2, IL-4, IL-5, IL-6, IL-9, IL-10, IL-12p70, IL-17A, and TNF- α) and chemokine (GM-CSF) were quantified with the Human MACSPlex Cytokine 12 Kit (Miltenyi Biotec) according to the manufacturer’s instructions. Samples were acquired on FACSVerse^™^cytometer (BD Biosciences) and analyzed with FlowLogic-v8 (Inivai Technologies).

### SARS-CoV-2 antibodies

The S1/S2-specific IgG were quantified with LIAISON SARS-CoV-2 S1/S2 IgG (DiaSorin) according to the manufacturer’s instructions, and expressed as IU/ml. The RBD-specific antibodies (i.e. IgM, IgA, IgG1 and IgG3) were determined by an in-house ELISA and expressed as area under the curve (AUC). Briefly, high-binding 96-well plates (Greiner Bio-One) were coated with 3 µg/ml of recombinant SARS-CoV-2-RBD (Creative Diagnostics) and incubated overnight at 4 °C. After 1 h blocking with PBS-2% BSA at 37 °C, plasma was serially diluted in duplicates, and incubated for 2 h at 37 °C. The following biotinylated antibodies were used: goat anti-human kappa and lambda light chain for total antibodies (Bethyl Laboratories, Inc.), rabbit monoclonal anti-human IgM and IgA (Abnova), mouse anti-human IgG1 (BD Biosciences) and IgG3 (Southern Biotech); followed by avidin-HRP (ThermoFischer Scientific) for 30 min at RT. The detection was carried out with 1 × 3,3’,5,5’-Tetramethylbenzidine and quenched with 1 M H_2_SO_4_. In each run, a plasma sample collected before the SARS-CoV-2 era was included. Additionally, the RBD-specific monoclonal antibody (Human Anti-SARS-CoV-2 Spike RBD Monoclonal antibody, Creative Diagnostics) was included as positive control for total RBD antibodies detection. Optical density (OD) was measured with Tecan SunriseTM at 450 and 620 nm.

### Antibody dependent cellular cytotoxicity (ADCC) assay

Parental CEM.NKr CCR5 + cells were mixed at a 1:1 ratio with CEM.NKr-Spike cells, stained for viability (AquaVivid; Thermo Fisher Scientific) and a cellular dye (cell proliferation dye eFluor670; Thermo Fisher Scientific) and subsequently used as target cells. Overnight rested PBMCs were stained with another cellular marker (cell proliferation dye eFluor450; Thermo Fisher Scientific) and used as effector cells. Stained effector and target cells were mixed at a 10:1 ratio. Plasma from COVID-19 + individuals (1/500 dilution) was added to the appropriate wells. Plates were centrifuged for 1 min at 300 *g*, and incubated at 37 °C for 5 h before being fixed. Since CEM.NKr-Spike cells express GFP, ADCC activity was calculated as follow: [(% of GFP + cells in Targets plus Effectors)—(% of GFP + cells in Targets plus Effectors plus plasma)]/(% of GFP + cells in Targets) × 100 by gating on transduced live target cells (Representative dot plots in Supplementary Fig. 5). Samples were acquired with LSRII cytometer (BD Biosciences) and data analysis performed using FlowJo v10.5.3 (Tree Star).

### Neutralization assay

293 T cells were transfected with the lentiviral vector pNL4.3 R-E- Luc (NIH AIDS Reagent Program) and a plasmid encoding for D614G Spike glycoprotein. Two days post-transfection, cell supernatants were harvested. For neutralization assay, 293 T-ACE2 target cells were seeded at a density of 1 × 10^4^ cells/well (Perkin Elmer) 24 h before infection. Pseudoviral particles were incubated with plasma dilutions (1/50; 1/250; 1/1250; 1/6250; 1/31,250) for 1 h at 37 °C and added to the target cells followed by 48 h incubation at 37 °C. Cells were lysed with 30 µL of passive lysis buffer (Promega) followed by one freeze–thaw cycle. An LB942 TriStar luminometer (Berthold Technologies) was used to measure luciferase activity after the addition of 100 µL of luciferin buffer and 50 µL of 1 mM d-luciferin potassium salt (Prolume).

### Immunophenotyping of PBMCs

1.5 × 10^^6^ of thawed PBMCs were plated in complete RPMI containing 10% human serum supplemented with 1% Penicillin–Streptomycin–Glutamin. Overnight-rested PBMCs were stained with the appropriate antibodies for 20 min at 4 °C in the dark and acquired using FACSVerse™cytometer (BD Biosciences). Dead cells were labeled using ViobilityTM Fixable Dye (Miltenyi Biotec). Antibodies used were: CD4-APC-Vio770, CD8-APC, HLA-DR-VioBlue, CD38-PE-Vio770, Granzyme B-PE and Perforin-FITC (Miltenyi Biotec). (Representative plots are shown in Supplementary Fig. 6). Data were analyzed using FlowJo 10.7.2 (BD Biosciences).

### Intracellular cytokine staining assay

Overnight-rested PBMCs were stimulated for 5 h with a pool of 15-mer peptides (1 μg/ml) covering the immunodominant sequence domain of the Spike (S), the complete sequence of the Nucleocapsid (N), or the complete sequence of the Membrane (M) proteins (PepTivator SARS-CoV-2, Miltenyi Biotec). Phorbol myristate acetate (25 ng/ml) and ionomycin (1 µg/ml) were used as positive control, negative controls were left untreated. Brefeldin A (1 mg/ml) was added after 1 h. Cells were harvested and stained for surface markers 20 min at 4 °C in the dark; after paraformaldehyde fixation, cells were permeabilized with 0.2% Saponin and stained for intracellular cytokines for 30 min RT. Antibodies used were: CD4-APC-Vio770, CD8-PerCP-Vio700, IL-17A-FITC, IL-4-PE, TNF-A-PE-Vio770, IFN-γ-VioBlue, IL-2-APC (Miltenyi Biotec). Dead cells were labeled using Viobility Fixable Dye (Miltenyi Biotec), and a total of 500,000 event were acquired using FACSVerse^™^cytometer (BD Biosciences). Representative plots are shown in Supplementary Fig. 6. Unspecific activation in unstimulated controls was subtracted from stimulated samples to account for specific activation. T-cells polyfunctionality was assessed by using the Boolean gating to identify single-, dual, triple cytokine-producing SARS-CoV-2-specific Th1 cells, whereas the analysis was performed with SPICE version 6.0. The magnitude of cytokine produced by a given subset was evaluated with an integrated median fluorescence intensity (iMFI) calculation by multiplying the frequency of Single cytokine, Dual cytokine, and Triple cytokine producing SARS-CoV-2 specific Th1 cells with the MFI for a given cytokine^37^.

### Statistics

Mann–Whitney *U* test was used for comparisons between groups for continuous variables (expressed as median, interquartile range, IQR); Fisher exact test for categorical variables (expressed as percentages). Spearman’s correlation was used to correlate viremia, expressed as log_10_RNA copies/ml, and immunological markers. Data were analyzed with GraphPad Prism 9.2.0.

## Supplementary Information

Supplementary Figures.

## Data Availability

Data that support the findings of this study are available upon reasonable request to the corresponding author.
