# Supplementary material for: Association between SARS-CoV-2 RNAemia and dysregulated immune response in acutely ill hospitalized COVID-19 patients
Source: Sci Rep. 2022 Nov 16;12:19658. doi: 10.1038/s41598-022-23923-1 (PMC9667450; doi:10.1038/s41598-022-23923-1)
Supplement: Supplementary file 1 — Supplementary Figures. [file 41598_2022_23923_MOESM1_ESM.pdf]

Title: Association between SARS-CoV-2 RNAemia and dysregulated immune response in acutely ill hospitalized COVID-19 patients

Authors: Roberta Rovito[1], Valeria Bono[1], Matteo Augello[1], Camilla Tincati[1], Federica Mainoldi[2], Guillaume Beaudoin-Bussières[3,4], Alexandra Tauzin[3,4], Silvia Bianchi[5], Mohamad Hadla[1], Vaibhav Yellenki[1], Antonella d'Arminio Monforte[1], Stefano Casola[2], Elisa Borghi[5], Andrés Finzi[3,4], Giulia Marchetti[1§]

Affiliations:

[1]Clinic of Infectious Diseases, Department of Health Sciences, ASST Santi Paolo e Carlo, University of Milan, Italy;

[2]The FIRC Institute of Molecular Oncology (IFOM), Milan, Italy;

[3]Centre de recherche du CHUM (CRCHUM), Montréal, QC H2X 0A9, Canada;

[4]Département de Microbiologie, Infectiologie et Immunologie, Université de Montréal, Montréal, QC H2X 0A9, Canada

[5]Microbiology and clinical Microbiology, Department of Health Sciences, ASST Santi Paolo e Carlo, University of Milan, Italy.

[§] **Corresponding author:** Giulia Marchetti, M.D., Ph.D, Clinic of Infectious Diseases, Department of Health Sciences, University of Milan, San Paolo Hospital, Milan, Italy, Via di Rudini' 8, 20142, Milan, Italy, Phone: +390281843046; Fax: +390281843054; Email: giulia.marchetti@unimi.it

Supplementary Figure 1. Correlation between SARS-CoV-2 viremia and nadir P/F

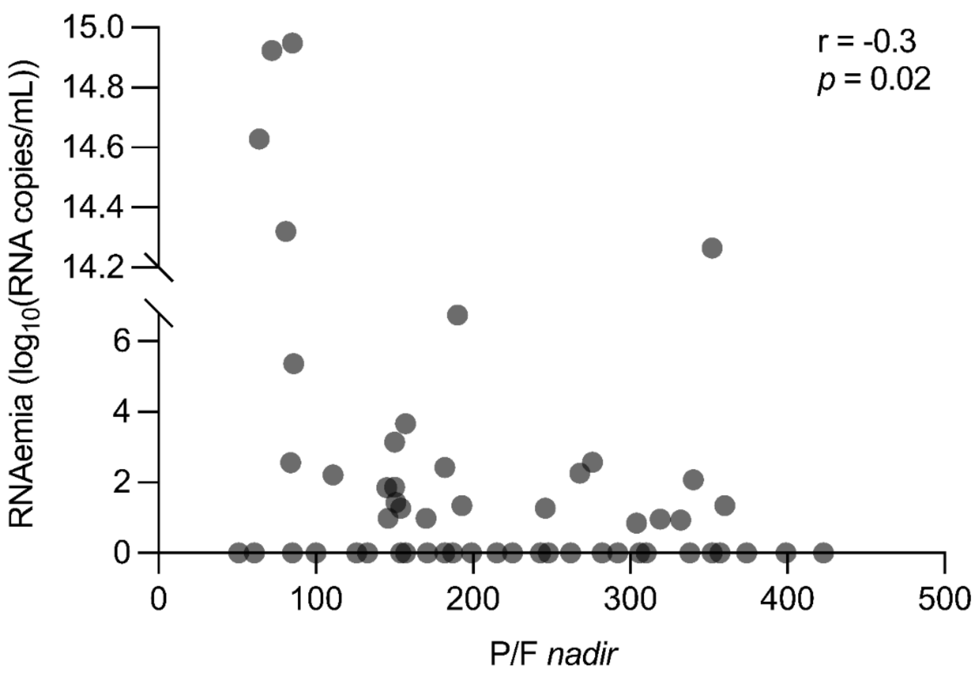

Spearman's correlation between SARS-CoV-2 viremia, expressed as log_{10}(RNA copies/ml), and nadir P/F.

Supplementary Figure 2. Heatmap of correlation

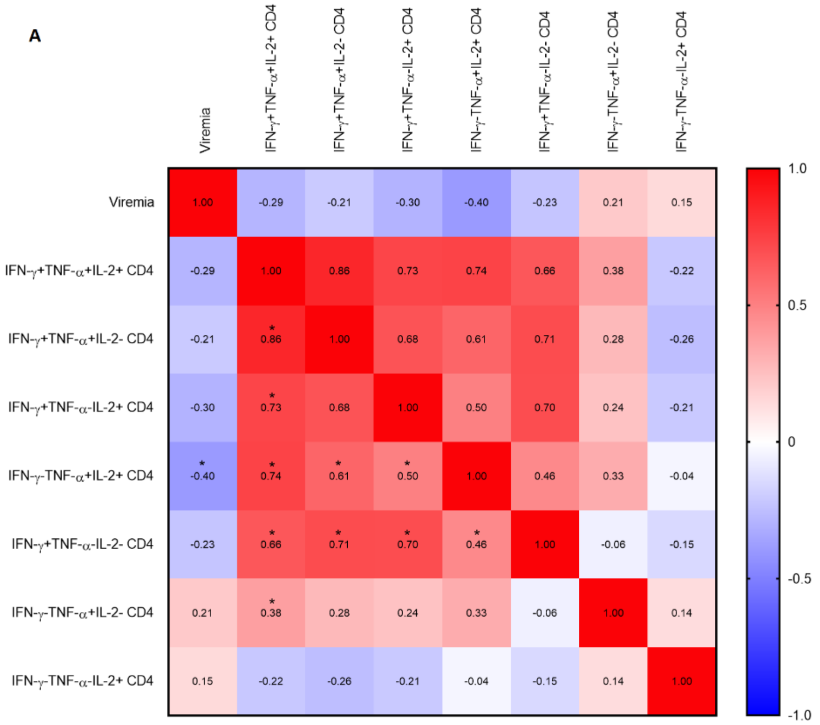

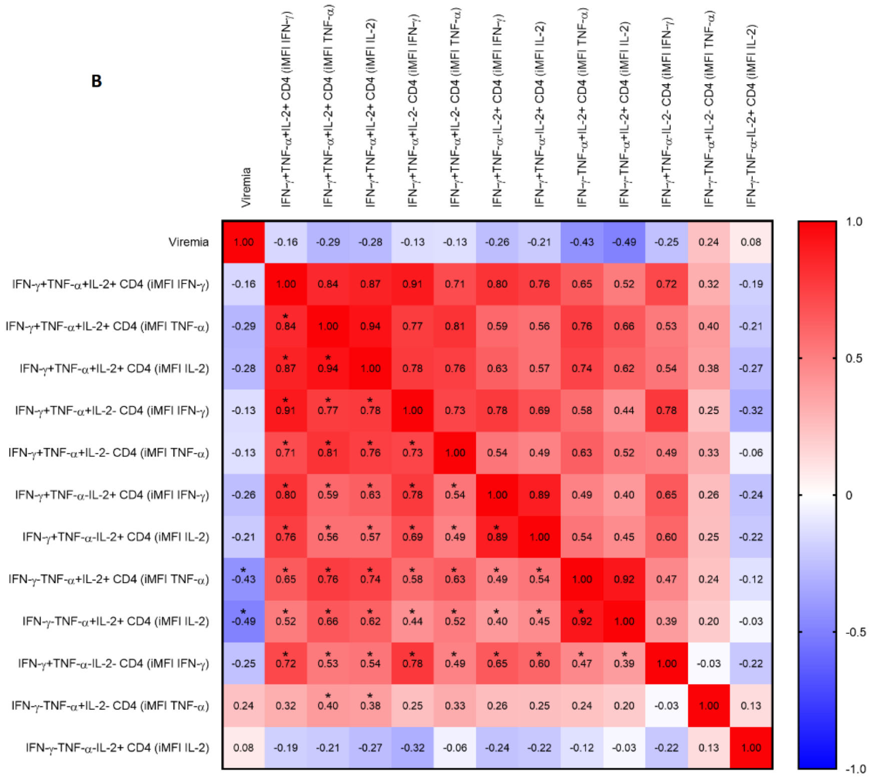

2A) Heatmap of correlations between SARS-CoV-2 viremia polyfunctional SARS-CoV-2-specific T cells. **2B)** Heatmap of correlations between SARS-CoV-2 viremia and iMFI of polyfunctional SARS-CoV-2-specific T cells.

Supplementary Figure 3. Th2 and Th17 skewness of SARS-CoV-2 specific CD4 Th1 cells

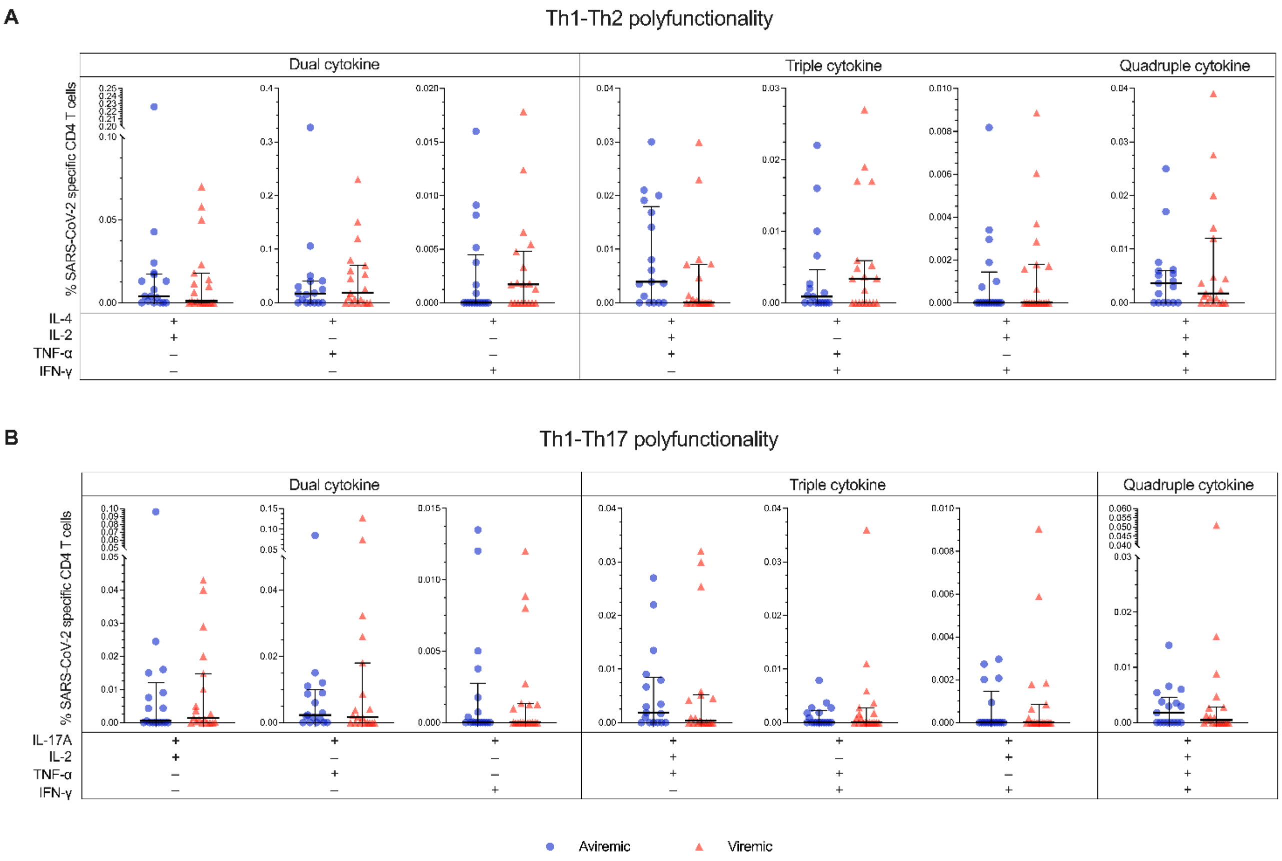

3A) Frequencies of SARS-CoV-2 specific CD4 Th1 cells producing IL-4 in aviremic and viremic COVID-19 patients (n=17 and n=19). **3B)** Frequencies of SARS-CoV-2 specific CD4 Th1 cells producing IL-17A in aviremic and viremic COVID-19 patients (n=19 and n=17). Median and interquartile range (IQR) are shown for each group of patients. Mann-Whitney U test, *statistical significance at p-value < 0.05.

Supplementary Figure 4. SARS-CoV-2 specific T cell cytokine production after challenge with individual pool of peptides

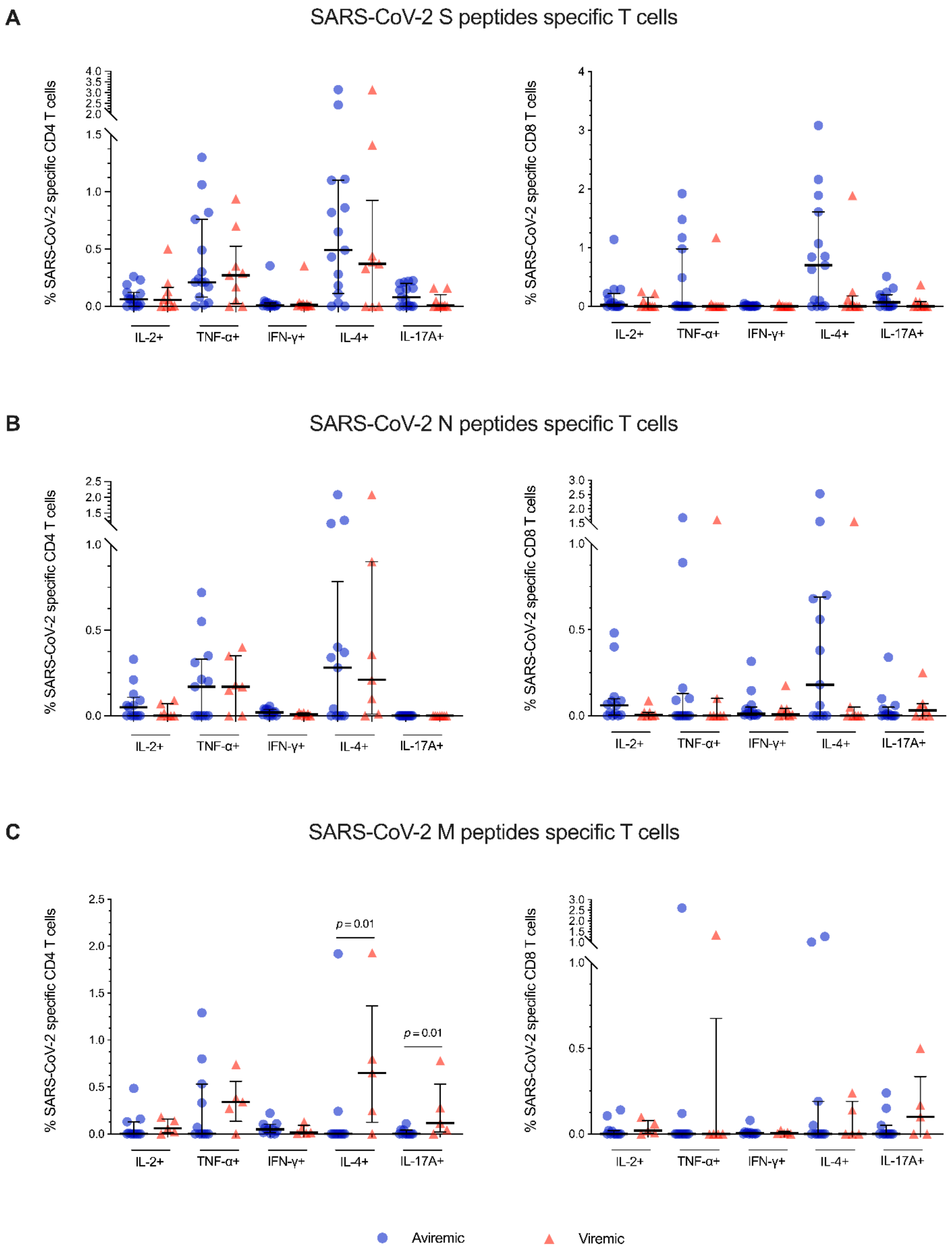

4A) Frequencies of IL-2, TNF-α, IFN-γ, IL-4 or IL-17A producing spike-specific CD4 and CD8 T upon PBMCs challenge for 5h with 1μg/ml of a pool of 15-mer peptides covering the immunodominant sequence domain of the spike protein (S) in aviremic and viremic COVID-19 patients (n=15 and n=9). **4B)** Frequencies of IL-2, TNF-α, IFN-γ, IL-4 or IL-17A producing nucleocapsid-specific CD4 and CD8 T upon PBMCs challenge for 5h with 1μg/ml of a pool of 15-mer peptides covering the entire sequence of the nucleocapsid protein (N) in aviremic and viremic COVID-19 patients (n=13 and n=7). **4C)** Frequencies of IL-2, TNF-α, IFN-γ, IL-4 or IL-17A producing membrane-specific CD4 and CD8 T upon PBMCs challenge for 5h with 1μg/ml of a pool of 15-mer peptides covering the complete sequence of the membrane protein (M) in aviremic and viremic COVID-19 patients (n=11 and n=11). Median and interquartile range (IQR) are shown for each group of patients. Mann-Whitney U test, *statistical significance at p-value < 0.05.

Supplementary Figure 5. Gating strategy for ADCC measurements

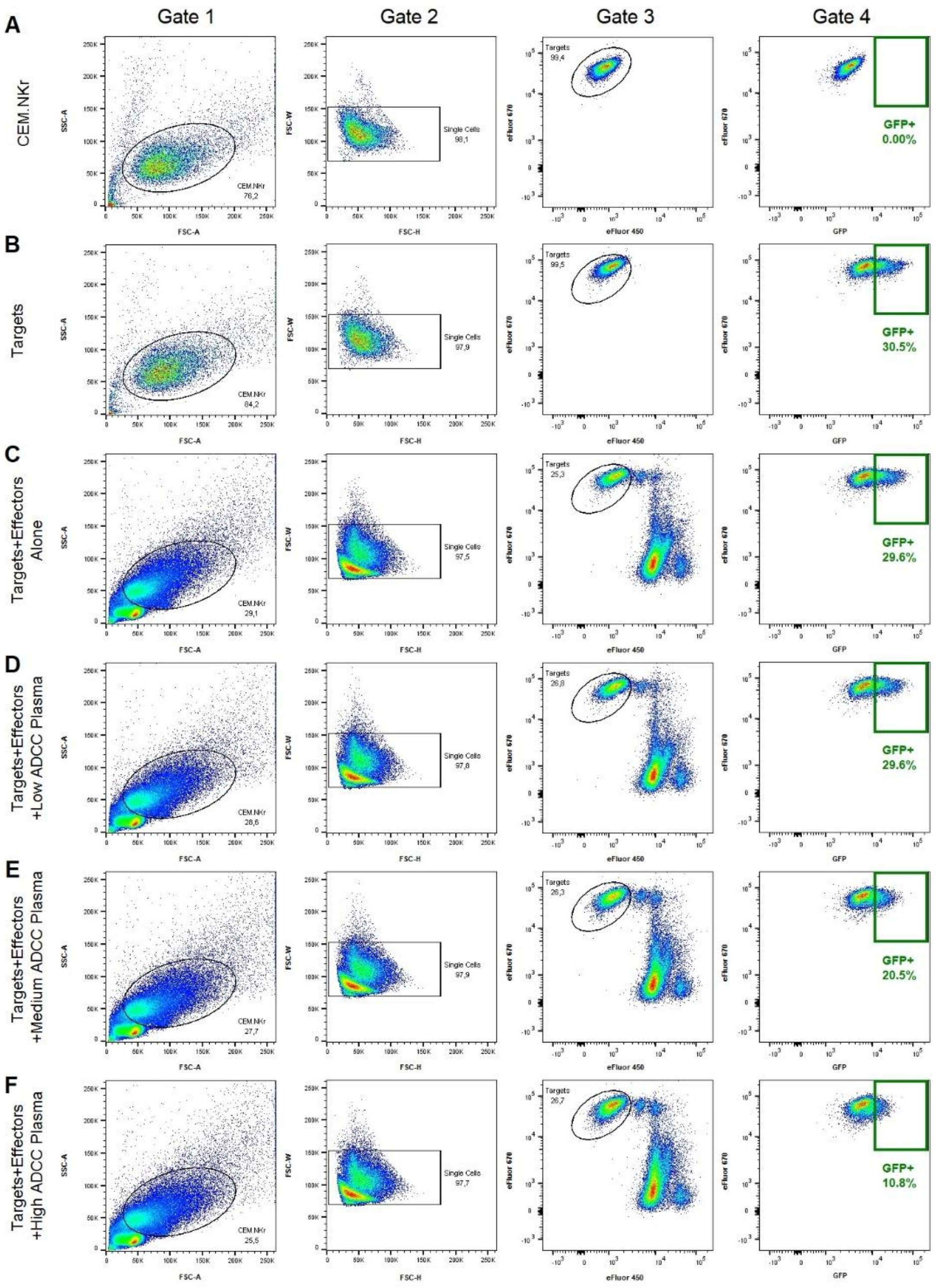

Target cells were identified according to cell morphology by light-scatter parameters (first column) and excluding doublets cells (second column). Cells were then gated on eFluor 670+ and eFluor 450- cells (thereby excluding the effector cells that were labeled with eFluor 450; third column). Last, the percentage of GFP+ target cells was used to calculate ADCC activity (fourth column). Examples of gating using **5A)** parental CEM.NKr cells, **5B)** a 1:1 ratio mix of parental CEM.NKr and CEM.NKr.Spike cells as target cells in absence or **5C)** in

presence of effector cells. Examples of plasma mediating low, medium and high ADCC activity were added to the target cells and effector cells in **5D)**, **5E)** and **5F)**, respectively.

Supplementary Figure 6. Gating strategy for Immunophenotyping and ICS

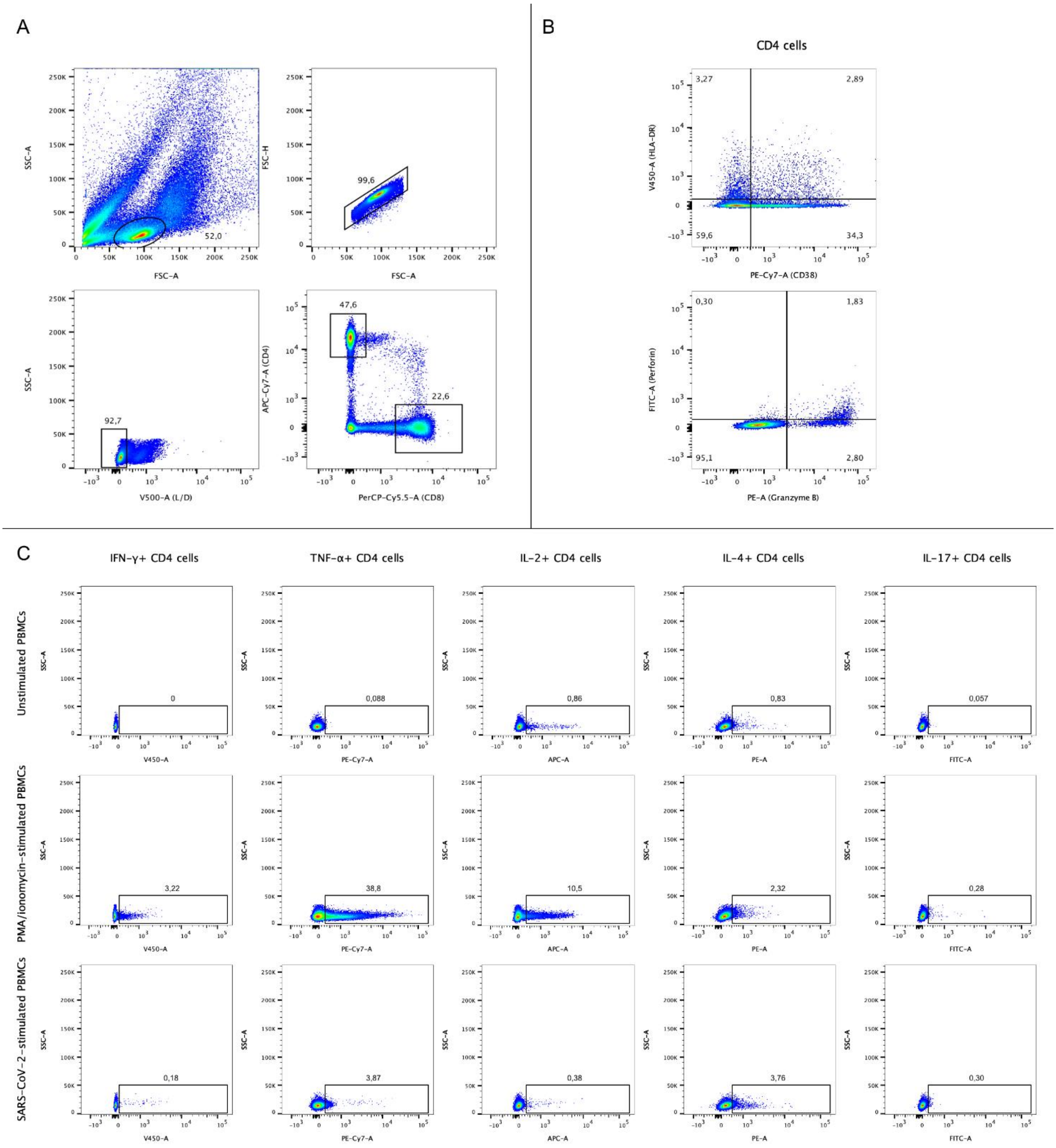

Gating strategy for identification of T cell immunophenotypes and SARS-CoV-2-specific T cells. **6A)** Lymphocytes, single and live cells were identified, and further divided into CD4 and CD8 T cells subsets. **6B)** Activated (HLA-DR+CD38+) or pro-cytolitic (PRF+GRZB+) CD4 T cells were identified, same gating strategy for CD8 T cells. **6C)** Identification of SARS-

CoV-2-specific cytokine-producing T cells: unstimulated, stimulated with positive control PMA/ionomycin and with SARS-CoV-2 peptide pool.
